# A decentralized privacy-preserving XR system for 3D medical data visualization using hybrid biometric cryptosystem

**DOI:** 10.1038/s41598-025-08784-8

**Published:** 2025-08-05

**Authors:** Shreyansh Sharma, Debasis Das, Santanu Chaudhury

**Affiliations:** 1 Department of Computer Science and Engineering, Indian Institute of Technology Jodhpur, Jodhpur, India; 2 Department of Electrical Engineering, Indian Institute of Technology Delhi, New Delhi, India; 3 Department of Computer Science and Engineering, NIIT University, Neemrana, Rajasthan, India

**Keywords:** 3D medical data visualization, XR, HBC, Redactable blockchain, Privacy-preserving healthcare, Brain imaging, Computer science, Biomedical engineering

## Abstract

In the era of digital healthcare, accurate and secure 3D visualization of medical data is critical for collaborative surgical planning. Traditional centralized systems suffer from security vulnerabilities and lack of depth cues necessary for accurate visualization of complex anatomy. We present a decentralized Extended Reality (XR)-based framework integrating a Hybrid Biometric Cryptosystem (HBC), hierarchical redactable blockchain, and InterPlanetary File System (IPFS)-based storage to address these limitations. The HBC combines leveled Homomorphic Encryption (HE) and Fuzzy Vault (FV) schemes for privacy-preserving multimodal biometric authentication. A hierarchical blockchain ensures tamper-resistance, consensus-based redactions, and secure access control. Photorealistic, spatially registered 3D models of brain MRI data are rendered in Augmented Reality (AR) and Mixed Reality (MR), enabling intuitive surgical planning. Edge caching accelerates data retrieval, enabling real-time interaction. Real-world deployment on Android and HoloLens 2 platforms demonstrates the clinical utility and robustness of the proposed framework. Security analysis confirms resistance to security threats such as replay, spoofing, etc, and unauthorized redactions. We achieve Equal Error Rates (EER) of 0.53% in AR and 0.68% in MR environments, with average authentication latency under 530 ms. A structured user study involving 40 clinicians confirms the system’s clinical utility, usability, and compliance with GDPR (General Data Protection Regulation) and HIPAA (Health Insurance Portability and Accountability Act) regulations. Therefore, the proposed framework offers a scalable, secure, and immersive platform for collaborative medical data visualization in digital healthcare.

## Introduction

The rapid evolution of immersive technologies is transforming digital healthcare, particularly in medical imaging and diagnostics. XR Technologies such as AR and MR can enable medical professionals to visualize and interact with 3D anatomical structures, such as brain MRI scans, and enable surgical planning in ways that traditional methods cannot^1–4^. Since the precision of planned surgery is critical to a patient’s life, the clarity, accuracy, and interactivity of visualized medical data cannot be compromised. This highlights the need for a high-resolution, 3D, and interactive visualization platform for patient medical data. Moreover, pre-operative planning of complex surgeries often necessitates collaboration of medical experts across different geo-locations by accessing and analyzing patient medical data in real time. However, relying on traditional protocol for remote access of medical data for sensitive applications such as medical data visualization raises concerns regarding data security & privacy, and breach of regulations like GDPR^5^.

Traditional medical data systems are centralized in nature, which makes them prone to unauthorized access, security breaches, and lack of transparency^6^. Moreover, relying on a single node for storage of a patient data creates a single point of failure, increasing risks of information leakage, attacks, reduced availability, and slower response times. Furthermore, the sensitive nature of medical data necessitates strict security and privacy guarantees, especially when accessed remotely over networks. These challenges are further intensified when considering cross-border collaborations, where regulatory compliance adds another layer of complexity to data sharing and access control. Therefore, a system designed to support remote collaborative surgical planning through immersive technologies must not only deliver seamless, high-resolution 3D visualization but also ensure secure, authenticated, and privacy-preserving access mechanisms.

In this work, we propose a hybrid framework that addresses these challenges by integrating leveled HE with a modified FV scheme and a hierarchical redactable blockchain architecture for secure and efficient XR-based 3D medical data visualization. Leveled HE enables computations on encrypted data for user registration and authentication without exposing sensitive information. The modified FV scheme complements this by securely storing biometric templates. The hierarchical blockchain architecture is built on the principle of redactability, where the parent layer performs biometric authentication of users accessing 3D medical data, and the child layer handles 3D visualization of patient medical data. Both layers store data in the IPFS. IPFS ensures data consistency across decentralized nodes through content-addressable storage. Each file is retrieved by a unique cryptographic hash, guaranteeing identical content across all nodes. Any modification results in a new content hash, which is recorded on the blockchain, enabling version control and traceability. Additionally, the smart contracts enforce collaborative access via a consensus-based access policy, where the access to patient medical data is granted if and only if all the designated experts authorize simultaneously. Otherwise, the framework does not render the visualization, thereby preserving data security & privacy. Similar to the collaborative access, the smart contracts enable safe redactability, where the need to modify/erase patient data may arise in scenarios such as successful cure or transfer of patient to another hospital, by enabling patient data erasure/modification if and only if all the designated experts approve simultaneously, thereby preventing malicious tampering. Furthermore, since 3D medical data is typically large, edge servers are employed to improve efficiency by caching frequently accessed data, enabling quick retrieval. If a patient’s data remains unchanged, its 3D visualization is fetched directly from the cache. Full retrieval from IPFS is only necessary when updates occur. Overall, a framework is developed for robust biometric authentication and efficient 3D medical data visualization while adhering to standard security regulations.

**Fig. 1:**
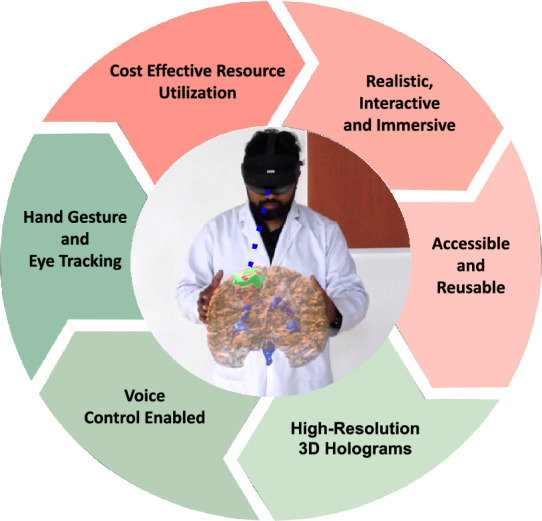
Illustrates the prominent features and advantages of the proposed XR application. It delivers a realistic, interactive, and immersive experience, simulating real-life scenarios for enhanced learning. Accessibility and reusability refer to the ability of using the modules repeatedly and using the same setup for other modules. High-resolution 3D holograms provide detailed anatomical visualization for better comprehension. The application enables cost-effective resource utilization by minimizing the need for physical robots in basic education. Voice control enables hands-free operation, enhancing user efficiency. Hand gesture and eye tracking facilitate intuitive interaction and precise control within the virtual environment.

The key features of the proposed framework are shown in Fig. 1, and the main contributions of this work are summarized as follows:

1. An XR-based framework is proposed for photorealistic, spatially registered 3D visualization of brain MRI data using both AR and MR, which enables remote and collaborative real-time surgical planning with intuitive interaction for understanding tumor structures.
2. A novel HBC is designed and integrated into the XR framework by combining leveled HE with FV techniques for secure and privacy-preserving user authentication, optimized for low-latency and real-time XR environments.
3. A hierarchical redactable blockchain architecture is integrated to provide secure and decentralized storage in IPFS, and also enable selective data modification or deletion through consensus-based authorization by medical experts, thereby ensuring GDPR compliance. Additionally, edge caching techniques are used to improve large 3D dataset retrieval and visualization efficiency.
4. Extensive experiments are performed to validate the proposed framework by real-time implementation on Android-based AR platform and Microsoft HoloLens 2 for MR. A structured user study involving 40 medical professionals, including neurosurgeons, radiologists, and surgical assistants is conducted by letting the professionals interact with the XR system for simulated surgical planning and tumor localization. Feedback collected in this survey reinforces the clinical relevance, usability, and effectiveness of the proposed framework.
5. A comprehensive security analysis demonstrates the robustness of the proposed framework against various threat models, confirming protection based on irreversibility, unlinkability, and regulatory compliance with standards such as GDPR and HIPAA.

The rest of the paper is organized as follows: Section “Related work” reviews related work in the fields of XR, biometric authentication, and blockchain technologies. Section “Threat model” summarizes the threats to the system. Section “Proposed methodology” presents the proposed hybrid framework, including its architecture and implementation. Section “Results and analysis” details experimental results and performance evaluation of the proposed framework. Section “Security analysis” discusses the security and privacy analysis of the framework, and Sect. “Conclusion” concludes the paper with future research directions.

## Related work

In the era of digital healthcare, medical practitioners are often required to take critical decisions on complex medical procedures and surgeries remotely. As a result, security & privacy of medical data has emerged as a critical concern in digital healthcare. To address this issue, various approaches have been proposed, each with distinct advantages and limitations. In^7^, the authors introduced a method that combines watermarking and blockchain to authenticate medical images. Watermarking embeds hidden data within images to prevent unauthorized modifications, while blockchain provides a tamper-resistant ledger for tracking access and changes. A mobile-based biometric authentication approach to secure Electronic Healthcare Records (EHRs) is introduced in^8^. This approach utilizes biometric data, such as fingerprints or facial recognition, to authenticate users accessing EHRs via mobile devices, thereby enhancing security by ensuring that only authorized individuals can access sensitive information. However, both^7,8^ primarily focuses on 2D images and do not extend to interactive 3D visualizations, limiting their applicability in complex surgical planning scenarios.

In the realm of the Internet of Medical Things (IoMT), a blockchain-enabled biometric security framework is proposed in^9^ to protect data from malicious attacks. While this framework enhances data security in wearable healthcare applications, it does not specifically address the challenges associated with 3D medical data visualization in XR environments. Similarly, the authors in^10^ developed a blockchain-integrated tamper-resistant framework for medical devices, aimed at ensuring data integrity and security. However, it does not incorporate biometric authentication, and does not explore the integration of immersive technologies to improve spatial awareness during surgical procedures. Consequently^9,10^, fall short in providing an intuitive interface for surgeons to interact with 3D models of patient anatomy.

On the other hand, a GDPR-compliant personal health record sharing mechanism using redactable blockchain and revocable IPFS is presented in Yeh et al.^11^ to ensure compliance with privacy regulations. This approach ensures data integrity and traceability while allowing modifications to comply with the ‘right to be forgotten’. However, it does not incorporate biometric authentication or immersive visualization techniques, which are crucial for secure and intuitive interaction with 3D medical data. While^12^ provides insights into biometric applications within XR, it does not propose a concrete framework combining biometric cryptosystems with blockchain for secure medical data management. Particularly biometric authentication enables secure and user-friendly access by leveraging unique physiological traits, like fingerprints or iris patterns^13,14^, protecting the biometric data is critical in such systems as security breaches lead to severe privacy violations. Cryptographic techniques like FV and HE have emerged as a promising solution by allowing error-tolerant authentication while ensuring privacy and unlinkability^15^. Moreover, the study does not address compliance with data privacy regulations such as GDPR. While decentralized biometric cryptosystems^16^ and immersive technologies^2,17^ have been developed individually, a framework that integrates XR with decentralized storage and access with biometric authentication has not been explored in the existing literature.

We propose a scheme which integrates XR-based immersive visualization with a HBC and a redactable blockchain architecture. This combination addresses the identified research gaps by providing a secure, intuitive, and GDPR-compliant framework for 3D medical data visualization and planning of surgeries. By enabling photorealistic, spatially registered 3D models and ensuring secure access through biometric authentication, our approach enhances clinical precision and workflow efficiency in surgical environments while meeting critical privacy and regulatory demands. To ensure the security and privacy of biometric templates and 3D medical data visualization, we consider the following threat model for the proposed framework.

**Fig. 2:**
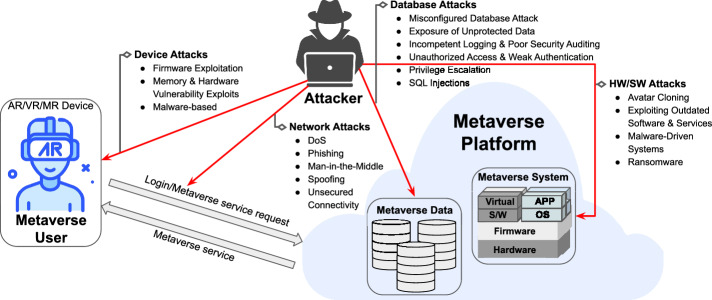
Threat model illustrating potential attack vectors across XR/Metaverse system layers including device-level, network, database, and application vulnerabilities. Key risks addressed include spoofing, phishing, malware injection, replay attacks, and SQL injection across various layers of the XR system architecture.

## Threat model

The potential threats targeted by the proposed framework are illustrated qualitatively in Fig. 2, and described as follows:

- Adversaries may attempt to gain unauthorized access to biometric and medical data in IPFS, thereby compromising confidentiality and integrity of sensitive data^18^.
- Attackers may attempt reconstruction of original biometric templates, leading to identity theft risks^15^.
- Adversary may establish linkability, causing issues such as profiling across multiple sessions and unauthorized cross-referencing of sensitive patient information.
- Adversaries may attempt malicious modification via unauthorized blockchain redactions, leading to compliance violations^19^.
- Replay attacks can be attempted to exploit biometric authentication data and undermine reliability.
- Man-in-the-Middle (MitM) attacks on data transmission may compromise security and privacy by interception or modification of biometric data, patient records, etc.
- Adversaries may leverage Denial-of-Service (DoS/DDoS) against blockchain and edge servers to disrupt or delay the availability of critical medical data visualization.

Each of these threats is addressed by the proposed framework, as detailed in the section below.

## Proposed methodology for XR

**Fig. 3:**
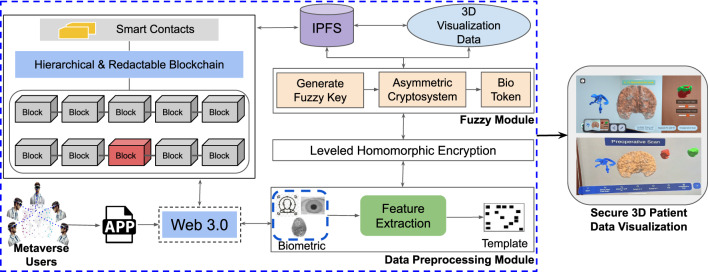
Proposed framework for Biometric-Based Secure 3D Patient Data Visualization, which integrates advanced biometric authentication with a hierarchical redactable blockchain and a hybrid encryption approach by combining leveled HE with a modified FV scheme to enable secure, efficient, and immersive XR-based visualization.

The architecture of the proposed framework is illustrated in Fig. 3. The process begins with a designated set of Metaverse users (medical experts) collaboratively accessing the system through a Web 3.0-enabled application. This interface serves as a gateway to access the decentralized visualization platform, which then employs biometric authentication for user authentication. The user biometric input is processed through a data Preprocessing module, where key features are extracted and transformed into a unique biometric template. This template is then passed into a fuzzy module, which generates a fuzzy key used for encryption and identification. It is important to note here that the framework grants access to the visualization if and only if all the designated experts authenticate and authorize simultaneously, as shown in Fig. 3.

Next, the fuzzy module produces a secure bio token using an asymmetric cryptosystem, which ensures that each session and user is uniquely verified. The generated bio token is then used within the leveled HE module, which performs secure computations on encrypted patient data without the need for decryption. This approach preserves data privacy while allowing computations and visualization to occur securely. The encrypted data is then logged on a hierarchical and redactable blockchain that supports smart contracts, as shown in Fig. 3. This blockchain infrastructure allows secure, tamper-resistant storage with the added capability of redaction under authorized conditions while simultaneously enabling collaborative investigation and planning of surgeries remotely. The patient data is ultimately stored in a decentralized manner using IPFS, ensuring that the data is distributed, immutable, and available across nodes, supporting the integrity and scalability of the system. Upon successful authentication and decryption, the relevant 3D patient data is retrieved and rendered within the XR environment. This immersive visualization allows medical practitioners to interact with secure, immersive, high-fidelity medical data models in an XR setting, enhancing both understanding and collaboration. The proposed architecture is implemented in multiple phases, as described below.

**Fig. 4:**
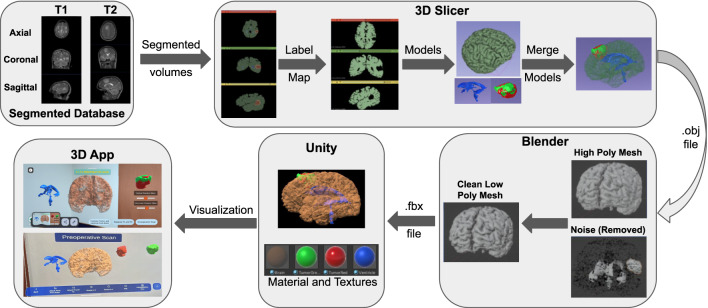
The process of XR-based 3D visualization: segmented MRI volumes from T1 and T2 scans are converted to label maps and 3D models using 3D Slicer. These models are merged, cleaned, and optimized in Blender to reduce polygon complexity. Final models are imported into Unity with textures for real-time visualization. The resulting application enables detailed AR/MR-based interactive exploration of brain anatomy and tumor data.

### Phase 1: XR-based 3D visualization system

This paper presents a novel framework for 3D visualization of patient data through XR-based interactive technology for brain MRI, as depicted in Fig. 4. This requires a high-resolution brain MRI scan from the patient. Then, the segmented structures and regions of interest are to be rendered in 3D, by incorporating advanced visualization techniques such as volume rendering and surface rendering. Subsequently, an XR application for 3D visualizations is developed such that the 3D renderings could be superimposed on a real-world environment by marker-less tracking methods for appropriate registration and alignment. Interactivity features including rotation, zooming, and measurement are implemented in the XR environment by an intuitive user interface with ‘pinch and stretch’ gesture. A view collaboration feature is also provided to increase the clinical utility of the system.

**Fig. 5:**
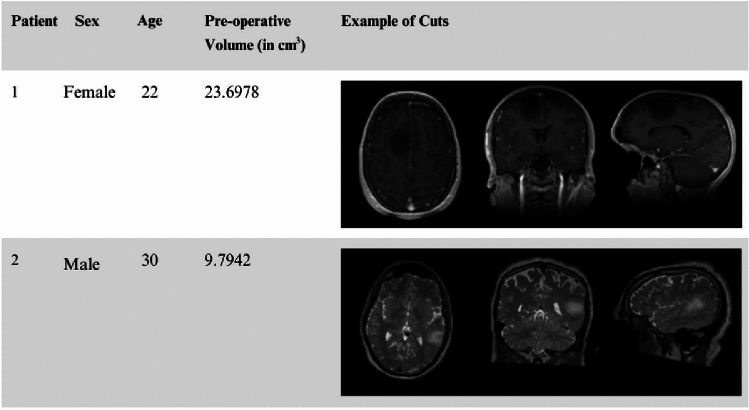
Sample demographic and imaging data from the ReMIND brain tumor MRI dataset^20^ used in the XR application.

**Fig. 6:**
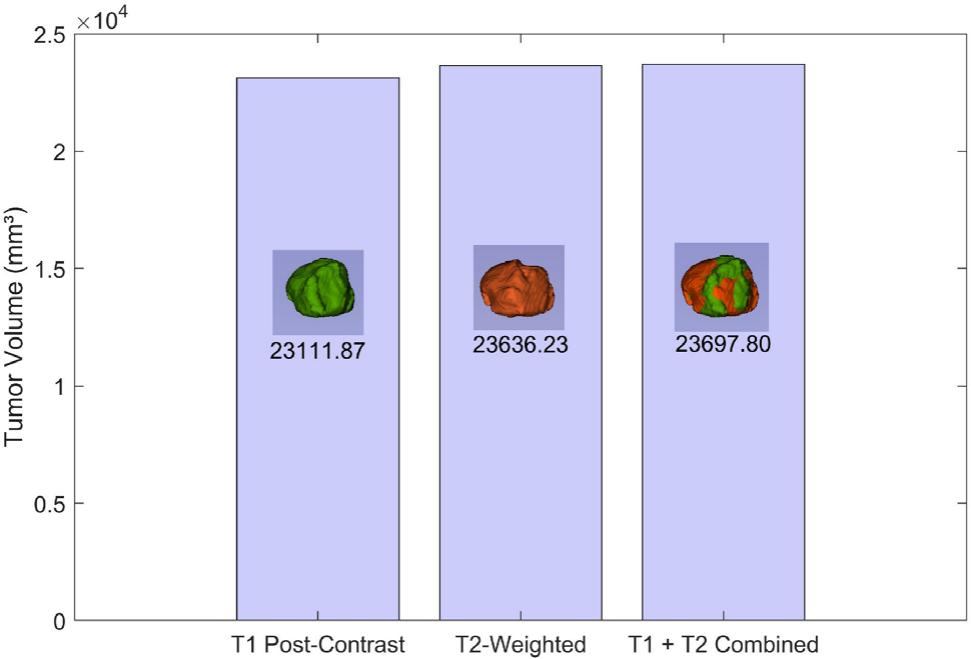
Difference between tumor shape and size when captured.

#### Database description

All brain tumors require resection with maximal safe surgical resection. Neuronavigation augments the ability of the surgeon to maximize this, however, surgery is an evolving process, and the information progresses into the past with the development of brain shift. More importantly, the high-grade gliomas are usually indistinguishable from the surrounding healthy brain tissue. Here we used the largest publicly available database^20^, ReMIND database. It consists of 123 consecutive patient datasets treated surgically with image-guided tumor resection in the AMIGO Suite at Brigham and Women’s Hospital (Boston, USA) between November 2018 and August 2022, with both iUS and iMRI. 9 of these cases were excluded because of corrupted or poor-quality data, leading to 114 cases. The exclusion criteria ranged from large iMRI artifacts (N = 1) to 3D iMRI corruption (N = 1) and poor quality of the intra-operative iUS before the opening of the dura (N = 7). The database consists of preoperative MRIs, for 114 consecutive patients who were surgically treated for brain tumors: 92 patients with gliomas, 11 with metastases, and 11 with other brain tumors. This increases the potential for research in brain shift and, in particular, image analysis, as well as neurosurgical training in the interpretation of iMRI. A sample of the data demographics is reported in Fig. 5 which includes patient id, gender, age, preoperative tumor volume and sample 2D DICOM slices.

#### Development workflow

The complete development workflow is presented in Fig. 4, including all the steps described below to obtain the required system.

***(a) Data preparation:*** The initial step involved acquiring segmented files in Nearly Raw Raster Data (NRRD) format. These files were obtained from the ReMind database and included information of brain structures like the cerebrum, the ventricles and the tumor. Each patient had two variations of scans: pre-operation and intra-operation. The pre-operation scans included two methods: T1-weighted post-contrast imaging and T2-weighted imaging, as shown in Figs. 4 and 6 . T1 post-contrast imaging enhances the visibility of the tumor by using a contrast agent, making it easier to delineate the tumor boundaries. In contrast, T2-weighted imaging is more sensitive to fluid content and generally shows a larger tumor volume due to its high sensitivity to the edema surrounding the tumor.

***(b) 3D visualization:*** The segmented files were then imported into the 3D Slicer software^21^, a comprehensive platform for medical image informatics. In 3D Slicer, the segmented files were loaded as volumetric data. A volume in this context refers to a 3D dataset where each voxel (3D pixel) holds intensity information, representing the scanned brain and its components. For each patient, this process was done separately for the pre-operation scans (both T1 post-contrast and T2-weighted) and the intra-operation scans showing the residual tumor.

- **Volume to label map conversion:** Within 3D Slicer, the volumetric data was converted into label maps, which is a type of image where each voxel is assigned a specific integer label representing different anatomical structures, such as the cerebrum, ventricles, and tumor. This conversion is crucial as it simplifies the delineation of different regions of interest for subsequent modelling.
- **3D model creation:** Every label map was processed to produce matching 3D surface models using the 3D Slicer. In this step, voxel-based label mappings were translated into polygonal meshes that faithfully capture the 3D structure of each anatomical component. The brain, ventricles, and cancer were all depicted in detail in the final models, as shown in Fig. 4. Three distinct 3D models were made for each patient: the T2-weighted pre-operation scan, the T1 post-contrast pre-operation scan, and the intra-operation scan that revealed the remaining tumors, as illustrated in Figs. 4 and 6 .

***(c) Model integration:*** The next stage was to combine the separate 3D models to create a complete brain model that featured the ventricles and tumor. Since this integration was carried out inside of 3D Slicer, it was guaranteed that the spatial relationships between the different structures would be precisely recorded and preserved. To enable in-depth comparison and analysis, distinct models were kept for the various scan variations (T1 and T2 pre-operation, and intra-operation).

***(d) Model optimization:*** This stage focuses on transforming the high-fidelity segmented anatomical models into lightweight, real-time compatible assets suitable for interactive XR rendering, while preserving anatomical accuracy and visual quality.

- **Polygon reduction:** The combined 3D model was saved in an OBJ file format by 3D Slicer. The OBJ file was then processed in Instant Meshes, a software tool designed for mesh simplification, in order to make it efficient for real-time use. In fact, one of the changes included lowering the polygon count both in terms of size and complexity, which is essential in optimizing the model for use in the field of AR.
- **Mesh refinement:** The low-poly model was then saved and processed in Blender^22^, a 3D modelling software available open-source. The final version of the model was refined in the Blender to keep only meshes necessary for the 3D object representation but ensure it was accurate anatomically and aesthetically. The model was then processed to an FBX file format, which is convenient while working with a graphics engine.

In the final rendering stage, the FBX models are imported into Unity, where physically-based materials, real-time shaders, and texture mapping are applied to achieve photorealistic visualization of brain structures, enhancing spatial perception and clinical interpretation in AR/MR environments.

### Phase 2: Redactable blockchain for data management

The proposed system utilizes a redactable blockchain architecture to meet the dual requirements of data integrity and compliance with data privacy regulations such as GDPR. Unlike traditional immutable blockchains, this system employs a stateful Chameleon Hash with Revocable Subkey (sCHRS)^23^ mechanism, enabling controlled modifications or redaction of data blocks. However, this feature is enabled via a consensus-based collaborative access through smart contracts, i.e., modifications or redaction is enabled if and only if all the designated experts approve simultaneously. This allows selective data updates while preserving the blockchain’s cryptographic properties and overall structural integrity. The statefulness of sCHRS ensures that each modification remains authorized and traceable, preventing malicious activities and supporting regulatory compliance^23^. The hierarchical blockchain consists of a parent layer for user identity and a child layer for medical data access. Redactions are enabled only when all designated experts provide authorization, enforced through smart contracts and validated using Chameleon Hash functions.

- **Redaction Mechanism:** Redactions are initiated by authorized users possessing revocable subkeys after receiving authorization by all the designated experts. The Chameleon Hash function verifies the legitimacy of these modification requests and cryptographically replaces the targeted data block while maintaining the original blockchain’s hash properties. This ensures that the blockchain remains consistent and tamper-proof, even after modifications.
- **Audit and Compliance:** Each redaction operation is logged as a transaction that records the authorized entity, type of change, and cryptographic proof. This mechanism maintains transparency and trust in the system, allowing independent nodes to verify modifications while supporting regulatory requirements such as GDPR’s “right to be forgotten.”
- **Permission Management:** Smart contracts automate access control by embedding permission rules that check user credentials and validate biometric authentication before allowing data modifications. This reduces the risk of unauthorized access and strengthens compliance with privacy laws.
- **Smart Contract Pseudocode for Access Control**
  To illustrate the collaborative authorization process in our redactable blockchain, the following pseudocode outlines the structure and logic of the smart contract used for validator-based access control.
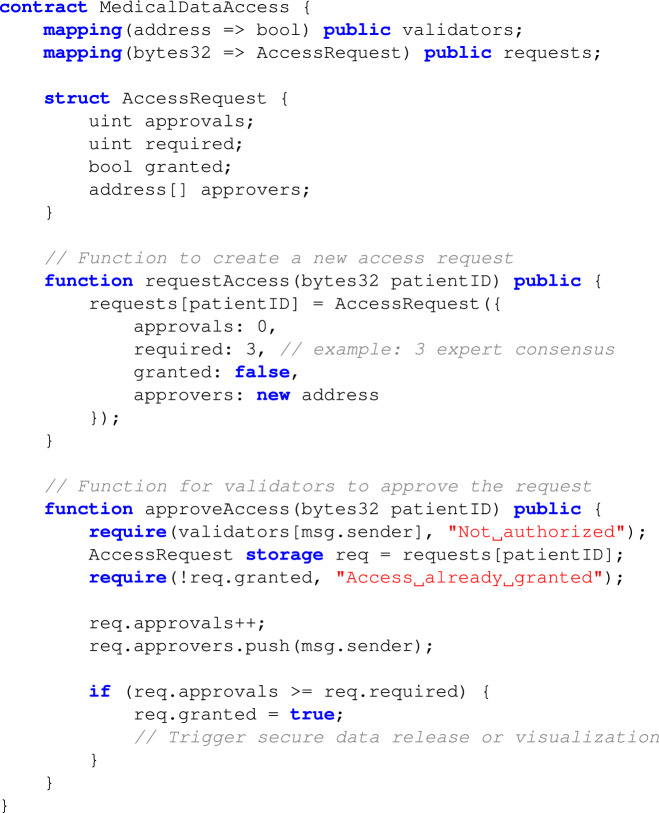
- **Redaction Transparency and Monitoring:** Every redaction is logged on the blockchain, providing an immutable audit trail. Smart contracts ensure that all authorized changes include cryptographic proofs, which can be reviewed by stakeholders to maintain transparency and traceability.

### Phase 3: HBC based authentication for XR

The proposed XR system incorporates a hybrid multimodal biometric authentication framework to ensure secure and reliable user identification while optimizing for real-time efficiency and simplicity. This phase combines leveled HE for critical operations and lightweight FV mechanisms for storage, achieving robust privacy, security, and computational feasibility. Blockchain technology is used for decentralized storage, ensuring irreversibility, unlinkability, and regulatory compliance. The multimodal biometrics are utilized in HBC for XR authentication to overcome the issues in single modality, i.e., enable robustness and security against malicious attacks. The HBC framework generates encrypted biometric tokens that are stored in IPFS, with their locations committed to the blockchain. During authentication, smart contracts verify user permissions and retrieve vaults securely, linking biometric verification with decentralized access control. Furthermore, the multimodal biometrics used for AR and MR are specifically chosen for the unique requirements of their immersive environment while ensuring user convenience and security. These modality choices were guided by clinical practicality: mobile-supported face and fingerprint recognition are suitable for AR settings, while gaze and PIN provide contactless, hygienic, and hands-free interaction required in sterile MR environments. Further details are explained in the following sections.

#### Authentication for AR

An Android application is developed to facilitate seamless interaction with the AR visualization system, as shown in Fig. 4. For secure access to the Android application for AR, face and fingerprint biometrics are selected as the multimodal biometrics to achieve efficient and user-friendly authentication process. Precisely, face recognition is chosen for its non-intrusive nature and ease of use in clinical and mobile settings, whereas fingerprint recognition is integrated as a supplementary biometric for its reliability and compatibility with existing mobile hardware. Building on prior work using these modalities^16^, the hybrid system proposed here integrates FV mechanisms and leveled HE for secure multimodal biometric authentication. To present hybrid the system for AR in detail, the following notations are introduced:

**Algorithm 1:**
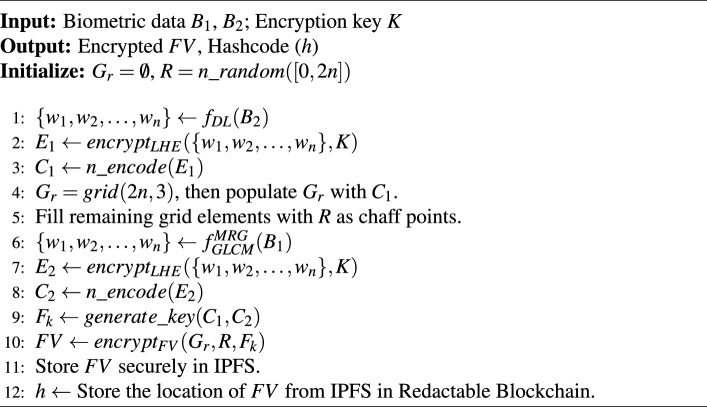
User Registration for AR

***Notations:*** Let $B_1$ (Biometric 1) and $B_2$ (Biometric 2) denote the dorsal hand and facial image biometrics, respectively. The following functions are used in the methodology: $n\_ random(p,q)$ generates *n* random integers in the range [*p*, *q*]; $f_{DL}(B)$ extracts features from *B* using a Deep Learning (DL) model; $f_{GLCM}^{MRG}(B)$ extracts features from *B* using Gray Level Co-occurrence Matrix (GLCM) and Minutiae Ridge Gabor (MRG) methods; $n\_ encode(E)$ encodes the encrypted weights $E_1$ to RS codes; *grid*(*p*, *q*) generates a $p \times q$ grid; $encrypt_{LHE}(X, K)$: leveled HE of sensitive data *X* using key *K*; $decrypt_{LHE}(X, K)$: Homomorphic decryption of sensitive data *X* using key *K*; $generate\_ key(C_1,C_2)$ generates a fuzzy key $F_k$ using RS codes $C_1$ and $C_2$; and $encrypt_{FV}(G_r, R, F_k)$: FV encryption of grid $G_r$, random numbers *R*, and fuzzy key $F_k$. Let $r_i$ and $c_i$ denote the $i^{th}$ row and column of $G_r$, respectively. *FV* and $\varnothing$ represent the FV and null vector, respectively. *dist*(*a*, *b*) denotes the Euclidean distance between *a* and *b* and $s_0>0$ is a certain threshold.

#### Proposed algorithm 1: user registration for AR

This algorithm presents an integration of leveled HE and the FV mechanism, where Leveled HE is used for feature encryption, ensuring privacy during biometric authentication, while the FV provides robust storage and retrieval of biometric data. First, a set of *n* integers, $R = n\_random([0, 2n])$, is randomly selected within the range [0, 2*n*]. DL techniques, such as CNNs, are employed for feature extraction from the facial image $B_2$, defined as $f_{DL}(B_2)$, resulting in feature weights $\{w_1, w_2, \ldots , w_n\}$. These extracted features are then encrypted via leveled HE using encryption key *K*, generating $E_1 = encrypt_{LHE}(\{w_1, w_2, \ldots , w_n\}, K)$. The encrypted weights are further encoded into RS codes to obtain $C_1 = n\_encode(E_1)$. Next, a $2n \times 3$ grid, $G_r = grid(2n, 3)$, is generated and populated with $C_1$, with remaining elements filled using randomly generated chaff rows for additional security. Similarly, minutiae-based feature extraction algorithms such as GLCM and MRG are applied to fingerprint data $B_1$, represented as $f_{GLCM}^{MRG}(B_1)$, to derive another set of feature weights $\{w_1, w_2, \ldots , w_n\}$. These are encrypted via leveled HE using the same encryption key *K*, producing $E_2 = encrypt_{LHE}(\{w_1, w_2, \ldots , w_n\}, K)$. The encrypted weights are subsequently encoded into RS codes to obtain $C_2 = n\_encode(E_2)$. Using $C_1$ and $C_2$, a fuzzy key $F_k = generate\_key(C_1, C_2)$ is generated. The FV is then constructed by encrypting the grid $G_r$ with the generated key and the randomly selected integers, yielding $FV = encrypt_{FV}(G_r, R, F_k)$. This FV is then stored into IPFS to ensure distributed and secure storage. Furthermore, the address of this storage in IPFS is stored in the readctable blockchain network to enable security via decentralized architecture.

**Algorithm 2:**
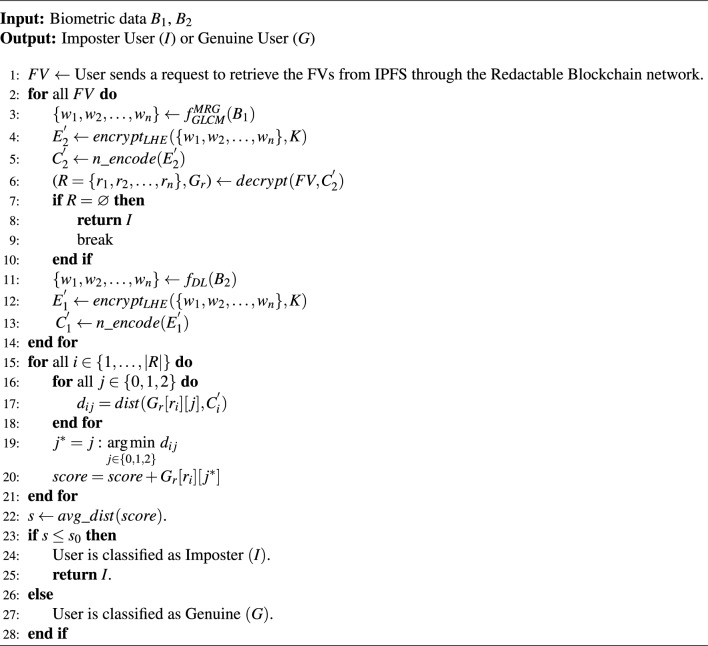
User Recognition for AR

#### Proposed algorithm 2: user recognition for AR

The user recognition framework for accessing the AR application is presented in Algorithm 2. The system first retrieves the stored *FV*s from IPFS through a redactable blockchain network. For each retrieved *FV*, the input biometric $B_1$ is processed using the function $f^{MRG}_{GLCM}$ to generate a weights $\{w_1, w_2, \dots , w_n\}$. These weights are encrypted into $E_2^{'} \leftarrow encrypt_{LHE}(\{w_1, w_2, \ldots , w_n\}, K)$ via leveled HE using Encryption key *K*. Subsequently, $E_2^{'}$ is encoded to obtain the RS codes ($C_2^{'}$), which are then used to decrypt the *FV* to produce *R*. If *R* is null after decryption, the user is immediately classified as an Imposter (*I*), and the algorithm terminates. Otherwise, the process continues to the second biometric $B_2$. $B_2$ undergoes feature extraction through function $f_{D_L}$ to produce another feature vector $\{w_1, w_2, \dots , w_n\}$. These weights are then encrypted into $E_1^{'} = encrypt_{LHE}(\{w_1, w_2, \ldots , w_n\}, K)$ via leveled HE using *K*. $E_1^{'}$ is subsequently encoded into RS codes ($C_1^{'}$). In the comparison phase, the algorithm iterates over each row $r_i$ in $G_r$ and computes the distances ($d_{ij}$) between the reconstructed grid entries $G_r[r_i]$ and $C_1^{'}$, for $j \in \{0, 1, 2\}$. The minimum distance $j^*$ for each feature $r_i$ is determined, and the corresponding grid scores $G_r[r_i][j^*]$ are aggregated to compute the total matching score. Once all features are processed, the algorithm evaluates whether the aggregated score exceeds a predefined threshold $s_0$. If the score is below the threshold, the user is classified as an Imposter (*I*). Otherwise, the user is authenticated and classified as Genuine (*G*).

**Algorithm 3:**
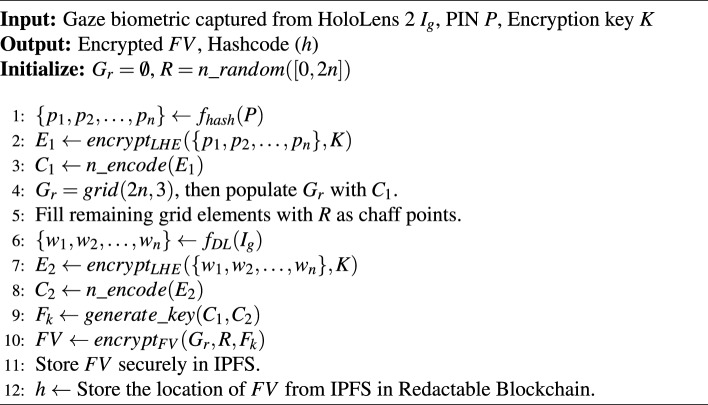
User registration for MR

#### Authentication for MR

The proposed HBC based MR authentication utilizes a combination of gaze-based behavioral biometrics and PIN authentication as the two biometric multimodalities. Gaze-based biometrics provide a contactless and hygienic authentication solution ideally suited for sterile medical environments, such as operating rooms. By eliminating the need for physical contact, it aligns naturally with clinical workflows and reduces the risk of contamination. Additionally, the integration of gaze biometrics within the proposed MR-based framework leverages the built-in eye-tracking capabilities of the HoloLens 2, eliminating the need for additional hardware and simplifying the deployment.

In particular, the gaze-based behavioral biometric leverages eye-tracking data such as fixation duration, saccades, and gaze trajectory, while PIN ensures an additional layer of security. In combination, these biometric modalities enable a non-intrusive, secure, and user-friendly authentication process. To present these authentication algorithms for MR in detail, we introduce the following notations in addition to those in the Sect. “Notations”:

***Notations:*** Let $I_g$ denote the gaze biometric captured from the eye-tracking system of HoloLens 2, and *P* denote the PIN input from the user. The function $f_{hash}(P)$ yields a 128 bit output using SHA-256 for *P*, which are used as feature set; and $f_{DL}(G)$ extracts gaze features using a DL model.

**Fig. 7:**
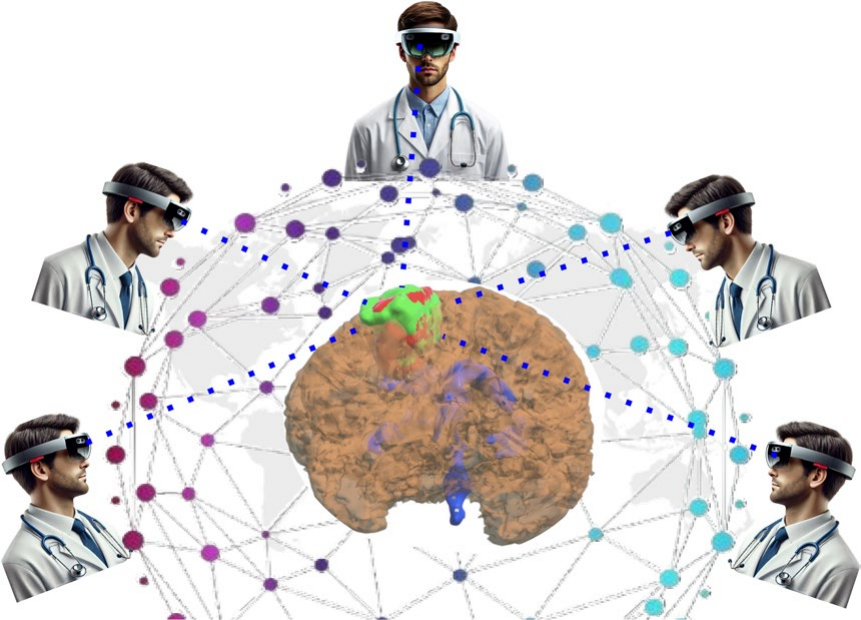
Collaborative Access of 3D Data visualization using app with HoloLens 2 from different geo-location.

#### Proposed algorithm 3: user registration for MR

The MR-based system employs a user-set PIN *P* and gaze biometric $I_g$ captured from the eye-tracking system of HoloLens 2. Firstly, a random set $R = n\_random([0, 2n])$ containing *n* integers randomly selected in [0, 2*n*] is generated. The feature weights $\{p_1, p_2, \ldots , p_n\}$ are extracted from the user-set PIN *P* using the function $f_{hash}(P)$, which yields a 128-bit output via SHA-256. These feature weights are encrypted via leveled HE using encryption key *K*, resulting in $E_1 \leftarrow encrypt_{LHE}(\{p_1, p_2, \ldots , p_n\}, K)$. The encrypted weights $E_1$ are then encoded to obtain the RS codes into $C_1 \leftarrow n\_encode(E_1)$. Subsequently, gaze features $\{w_1, w_2, \ldots , w_n\}=f_{DL}(I_g)$ are extracted using deep learning techniques via function $f_{DL}(I_g)$, and encrypted via leveled HE into $E_2 = encrypt_{LHE}(\{w_1, w_2, \ldots , w_n\}, K)$. The encrypted gaze weights are encoded into RS codes $C_2 \leftarrow n\_encode(E_2)$. Then, a $2n \times 3$ grid $G_r = grid(2n, 3)$ is generated and populated with elements of $C_1$, with the remaining elements filled with random integers to form chaff rows. A fuzzy key $F_k$ is generated as $F_k = generate\_key(C_1, C_2)$. The FV is then obtained as $FV=encrypt_{FV}(G_r, R, F_k)$, and stored in IPFS for distributed, efficient, and secure storage. Finally, the location of *FV* in IPFS is stored in the redactable blockchain network. Therefore, this algorithm for MR employs an integration of leveled HE and the FV mechanisms, as shown in Algorithm 3.

**Algorithm 4:**
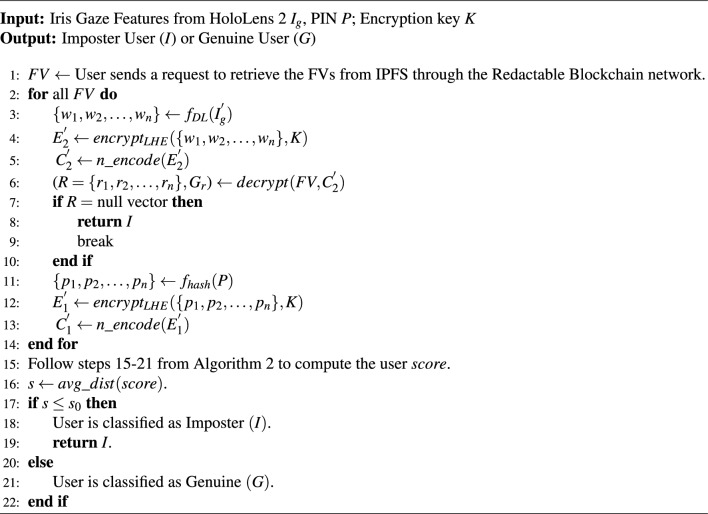
User Recognition for MR

#### Proposed algorithm 4: user recognition for MR

For accessing the MR visualization, Algorithm 4 presents the user recognition framework. Firstly, the stored *FV*s are retrieved from IPFS through a redactable blockchain network. For each retrieved *FV*, the weights $\{w_1, w_2, \dots , w_n\}=f_{DL}(I_g^{'})$ are derived using DL techniques from the gaze-based behavioral biometric $I_g^{'}$ of the user via eye-tracking system of HoloLens 2. These weights are encrypted into $E_2^{'} \leftarrow encrypt_{LHE}(\{w_1, w_2, \ldots , w_n\}, K)$ via leveled HE using Encryption key *K*. $E_2^{'}$ is subsequently encoded to RS codes ($C_2^{'}$) and used to decrypt the *FV* to generate *R*. If *R* is null after decryption, the user is deemed an Imposter (*I*), and the algorithm terminates. Otherwise, the algorithm proceeds to the second biometric modality, which is the PIN authentication for MR. The user enters their PIN (*P*) using a virtual gaze-controlled keypad. Another set of weights $\{w_1, w_2, \dots , w_n\}$ are then derived from *P* through function the $f_{hash}(P)$, which yields a 128 bit output using SHA-256. These weights are then encrypted into $E_1^{'} = encrypt_{LHE}(\{w_1, w_2, \ldots , w_n\}, K)$ via leveled HE using *K*. $E_1^{'}$ is subsequently encoded into RS codes ($C_1^{'}$). In the comparison phase, the algorithm iterates over each row $r_i$ in $G_r$ and computes the distances ($d_{ij}$) between the reconstructed grid entries $G_r[r_i]$ and $C_1^{'}$, for $j \in \{0, 1, 2\}$. The minimum distance $j^*$ for each feature $r_i$ is determined, and the corresponding grid scores $G_r[r_i][j^*]$ are aggregated to compute the total matching score. Once all features are processed, the algorithm evaluates whether the aggregated score exceeds a predefined threshold $s_0$. If the score is above the threshold, the user is authenticated and classified as Genuine (*G*). Otherwise, the user is deemed an Imposter (*I*).

In summary, the proposed multimodal authentication for MR combines gaze tracking with knowledge-based PIN authentication while leveraging Leveled HE and FV mechanisms for privacy preservation. The virtual PIN keypad for knowledge-based PIN authentication also offers the added advantage of Shoulder Surfing Resistance by randomizing layout to prevent observation attacks. Additionally, the proposed Algorithms 3 and 4 use the built-in HoloLens 2 sensors, maintaining high usability and security without requiring external hardware. Therefore, the Algorithms 3 and 4 provide secure access to MR visualization of medical data for users from different geo-locations, as shown in Fig. 7.

## Results and analysis

In this section, the performance of the proposed framework is validated using standard metrics through extensive experiments and user feedback analysis.

### Experimental setup

The experimental setup for the 3D visualization of tumor lesions in the affected brain consisted of high-performance computing hardware and an integrated software framework to support AR, MR, biometric processing, IPFS, and blockchain integration. In particular, an HP Elite Tower CPU was utilized for the 3D visualization, featuring a 12th Gen Intel Core i7-12700 processor (2.10 GHz, 12 cores), 32.0 GB RAM (31.7 GB usable), a 1 TB SSD for primary storage, an additional 500 GB HDD, and an Nvidia T1000 graphics card with 8 GB VRAM, running on Windows 11 Pro. The visualization was displayed on a Full HD HP Monitor M27ha. For application deployment and testing, a Pixel tablet was used, equipped with a 10.95-inch IPS LCD display, a Google Tensor G2 (5nm) chipset, an octa-core CPU (2$\times$2.85 GHz Cortex-X1, 2$\times$2.35 GHz Cortex-A78, and 4$\times$1.80 GHz Cortex-A55), and a Mali-G710 MP7 GPU. The device featured 8 GB RAM, 128 GB UFS 3.1 storage, and was powered by Android 14.

The previously generated FBX model was imported into Unity 2021.3 LTS, utilizing the ARCore SDK for AR-based visualization and interaction. In Unity, appropriate materials and textures were applied to the brain model to enhance visual distinction and realism. Furthermore, for the MR-based visualization, the application was deployed on Microsoft HoloLens 2, leveraging MRTK3 for an optimized MR experience. The system incorporated real-time biometric processing using Python, TensorFlow, and OpenCV to analyze gaze-tracking data captured from the HoloLens 2. Additionally, the implementation of blockchain-based security mechanisms involved Solidity-based smart contracts deployed on the Ethereum Testnet, with biometric data securely stored using IPFS. To ensure a robust and scalable approach, the system was trained and validated using the ReMIND Brain MRI Database^20^ for tumor visualization, along with biometric datasets comprising face and fingerprint data for authentication. Gaze-tracking data from the HoloLens 2 was further utilized to refine the interaction model. A user interface was developed within Unity to facilitate intuitive interaction with the 3D model, providing seamless navigation and real-time engagement.

### Software versions and URLs

The software tools and libraries used in this study to develop and implement the proposed XR framework include 3D Slicer (version 5.0.3, https://www.slicer.org) for medical image processing and segmentation, Instant Meshes (version 0.5, https://github.com/wjakob/instant-meshes) for mesh simplification, Blender (version 3.5, https://www.blender.org) for 3D model refinement, and Unity 2021.3 LTS (https://unity.com/releases/2021-lts) for application development. For augmented reality on Android devices, the ARCore SDK (version 1.37.0, https://developers.google.com/ar) was utilized. Mixed reality applications on the HoloLens 2 employed the Microsoft Mixed Reality Toolkit MRTK3 (version 3.0.0, https://learn.microsoft.com/en-us/windows/mixed-reality/mrtk-unity/). Biometric processing and authentication algorithms were implemented using Python 3.10 (https://www.python.org), with TensorFlow 2.12 (https://www.tensorflow.org) and OpenCV 4.7 (https://opencv.org) libraries.

**Fig. 8:**
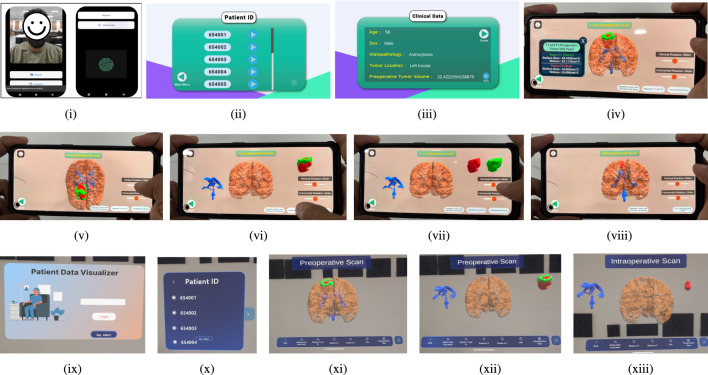
Workflow of the proposed XR-based application, which enables enhanced cognitive representation and surgical planning through immersive interaction. The process begins by secure biometric login, patient data access, and 3D brain tumor visualizations. AR and MR interfaces allow surgeons to view, rotate, and isolate anatomical structures across T1, T2, and intra-operative scans. AR is accessed via Android mobile (**i**–**viii**), while MR uses HoloLens 2 with gaze authentication (**ix**–**xiii**).

#### Interface details

The final application was built with a focus on usability, where each interactive model corresponds to one of three distinct cases: T1 post-contrast pre-operation, T2-weighted pre-operation, and intra-operative scans. This approach provides users with multiple cognitive representations of the tumor at various stages of its progression, as illustrated in Fig. 8. Here, (i) presents the user (surgeons) interface with a secure login screen for AR, identifying the user via their face and fingerprint biometrics. It is important to note that the framework grants access if and only if all users are authenticated and simultaneously authorize the visualization. Upon successful authentication, surgeons are directed to (ii), which displays a comprehensive list of patients identified by unique IDs and not their names, thereby maintaining confidentiality. On the next page, (iii) provides detailed patient information, including age, sex, histopathology data, tumor location, and preoperative tumor volume. In (iv), the surgeon then utilizes a tablet application to access an AR view of the brain with accompanied information of pre-operation scan, and the zoom-in and zoom-out functionality of AR interface. Tumor surface areas are distinctly marked, with T1 regions in green and T2 regions in red. Then (v) depicts interaction with the model through vertical and horizontal rotations. (vi) and (vii) highlight the ability to separate and visualize distinct anatomical structures within the AR environment, including the cerebrum, tumor, and brainstem. Finally, (viii) illustrates the intra-operative scan of the affected region, providing a comprehensive AR visualization tool for surgical planning and analysis.

Similarly, the user interface for MR visualization is shown in Fig. 8(ix), where the user is verified via gaze-based biometrics from HoloLens 2 and the pre-set PIN. Upon successful authentication, the surgeon is directed to the page displaying patient IDs in (x). The next page resembles (iii) for MR visualization as well, where the user is then directed to (xi), which provides 3D MR visualizations of pre-operative scans of affected brain. Subsequently, (xii) highlights the ability to separate and visualize distinct anatomical structures within the MR environment, including the cerebrum, tumor, and brainstem. Finally, (xiii) demponstrates the intra-operative scan of the affected region, providing a comprehensive MR visualization tool for surgical planning and analysis.

**Fig. 9:**
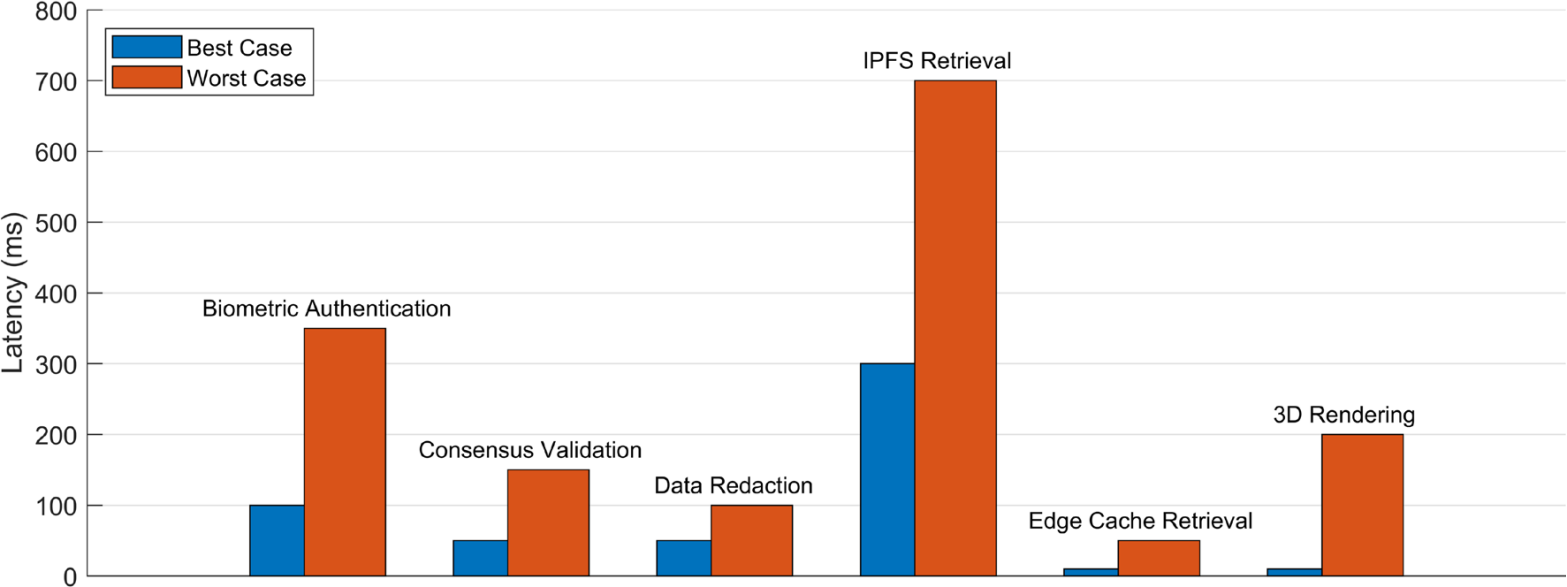
Performance metrics for decentralized framework.

### Performance metrics

Figure 9 presents a comparative latency analysis across six critical operations of the proposed framework that integrates biometric authentication, blockchain validation, data redaction, decentralized storage (IPFS), edge caching, and 3D rendering. The best-case scenario, represented by blue bars, reflects optimal operating conditions, characterized bylow network latency, efficient computation, and high cache availability. Under such conditions, biometric authentication requires approximately 100 ms, consensus validation takes around 50 ms, and IPFS retrieval completes in roughly 300 ms. Other processes such as data redaction and edge cache retrieval exhibit minimal latencies of about 50 ms and 10 ms respectively, while 3D rendering takes nearly 10 ms. In contrast, the worst-case scenario (shown in orange bars) accounts for elevated network delays, computational overheads, and data unavailability in cache, leading to increased execution times. Under these conditions, biometric authentication latency rises to $\tilde{3}50$ ms, consensus validation reaches 150 ms, and IPFS retrieval peaks at 700 ms. Data redaction may take up to 100 ms, edge cache retrieval 50 ms, and 3D rendering about 200 ms. Leveled HE introduces computational cost, but system-level optimizations like edge caching keep authentication latency under 530 ms. This performance is sufficient for real-time medical applications without impacting user experience. These values highlight the adaptability and resilience of the proposed framework, which successfully balances decentralization, security, and real-time performance across a range of operational environments.

**Fig. 10:**
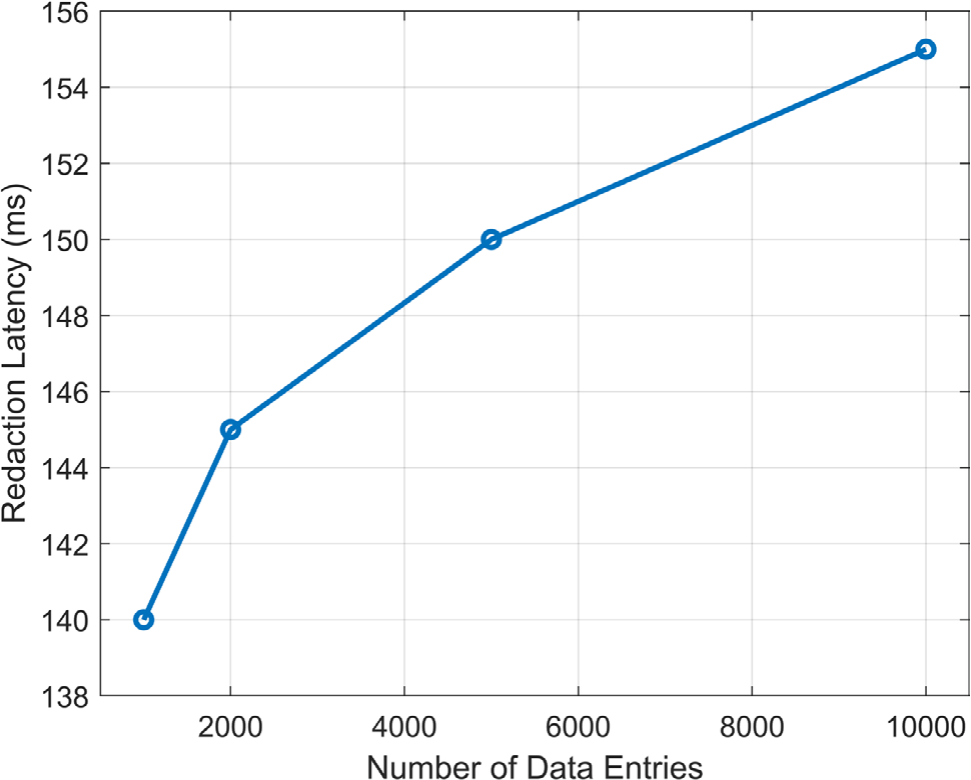
Redaction latency vs. number of data entries.

**Fig. 11:**
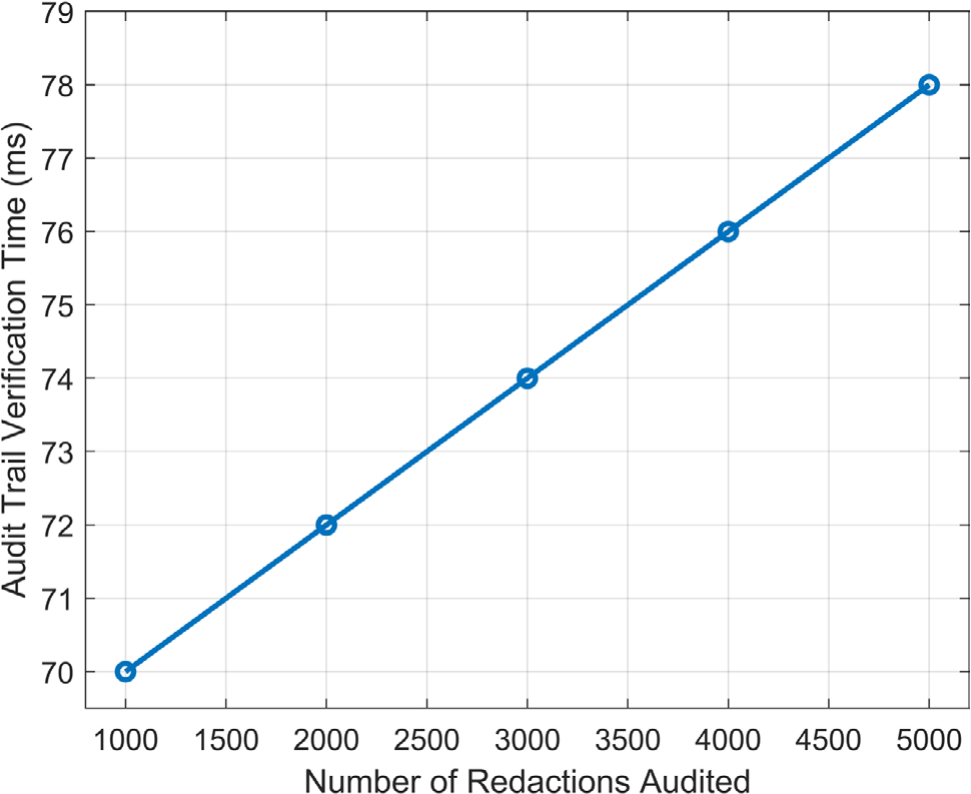
Audit trail verification time vs. number of redactions.

Furthermore, the performance of the proposed redactable blockchain system, based on sCHRS, was evaluated using on key metrics such as redaction latency, computational overhead, audit trail verification time, and compliance audit time, as shown in Figs. 10 and 11 . These metrics demonstrate the system efficiency in ensuring secure data management and regulatory compliance. The relationship between redaction latency and the number of data entries is shown in Fig. 10. For modifying 10,000 data entries, the average redaction latency was 150 ms with a standard deviation of ±12 ms, indicating that the system supports real-time data management operations without significant delays. For auditing 5,000 redactions, the Audit trail verification time averaged 75 ms with a deviation of ±8 ms, as illustrated in Fig. 11. This rapid verification ensures data integrity and supports regulatory compliance. Additionally, the compliance audit time for a full data compliance check was 1.5 s with a standard deviation of ±0.3 s, demonstrating the ability of the system to perform comprehensive evaluations efficiently. Additionally, the system records redaction actions in a transaction log, including details such as transaction IDs, action performed, authorized entity, timestamps, and proof of redaction in Table 1 to maintain transparency and accountability. This log also enhances traceability and facilitates regulatory audits. It is important to note here that a permissioned blockchain network has been employed in the proposed framework, ensuring access only to authorized users within the network, referred to as ‘Validators’ in Table 1.

**Table 1:** Consensus-based redaction transaction Log.

Transaction ID	Action performed	Authorized entity	Timestamp	Proof of redaction
0xf6a2bcd1	Patient data redaction	Validators	2025-01-03 09:12:45	Verified (Multi-signature)
0xe3b9cd78	Metadata update	Validators	2025-01-04 11:05:22	Verified (Multi-signature)
0x8d42af91	Patient record erasure	Validators	2025-01-06 14:23:10	Verified (Multi-signature)
0x9e1f77c3	Consent withdrawal logged	Validators	2025-01-08 10:45:33	Verified (Multi-signature)
0xa72bcd12	Visualization access approval	Validators	2025-01-10 16:02:18	Verified (Multi-signature)
0xb61ef8dd	Secure data update	Validators	2025-01-12 08:39:50	Verified (Multi-signature)
0xc8dfac34	Audit log entry added	Validators	2025-01-14 13:11:09	Verified (Multi-signature)

### Biometric authentication performance

The biometric authentication framework was evaluated based on the key performance metrics shown in Table 2, namely False Acceptance Rate (FAR), False Rejection Rate (FRR), EER, and authentication latency. In contrast to the single biometric modality (i.e., face, fingerprint, and gaze-biometric, respectively) the proposed AR authentication system that employs multimodal biometrics (face and fingerprint) achieves a significant improvement in the performance metrics as shown in Table 2, yielding a FAR of 0.27%, FRR of 0.81%, and an EER of 0.53, with an authentication latency of 530 ms. In the MR system, which utilized a combination of gaze-based biometric and PIN input for the multimodal biometrics, the results were comparable, with a FAR of 0.45%, FRR of 0.92%, and an EER of 0.68, with an authentication latency of 480 ms.

**Fig. 12:**
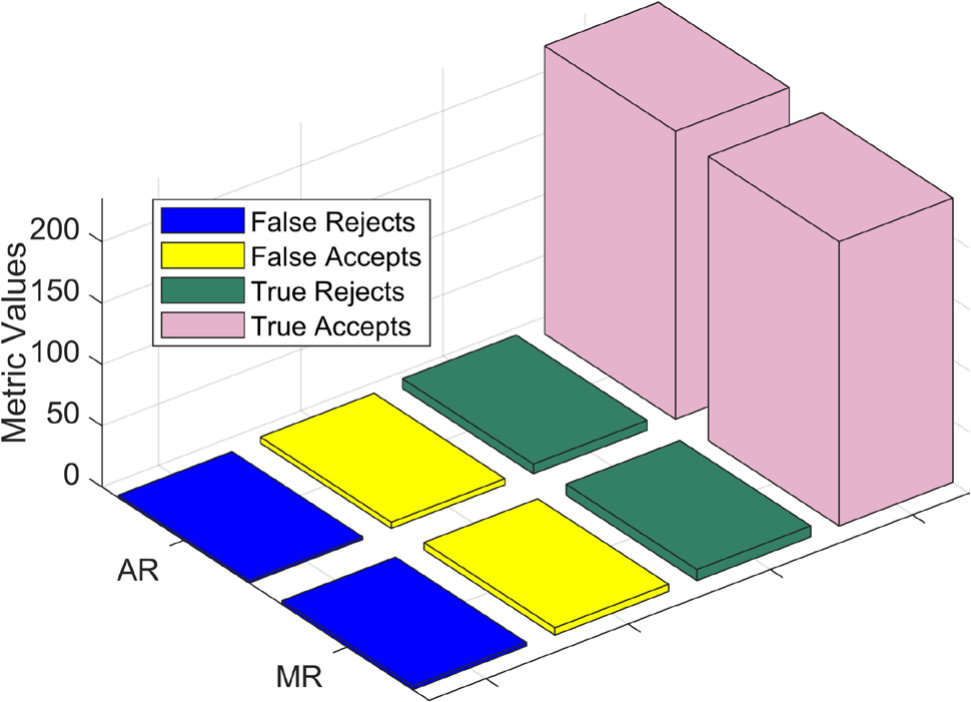
Comparative analysis of AR vs MR.

The comparative performance of the AR and MR systems is visually illustrated in Fig. 12, which presents a 3D bar graph summarizing the metric outcomes. The bars are categorized into the following 4 metrics: False Rejects, False Accepts, True Rejects, and True Accepts-across the two modalities (AR and MR). The Y-axis represents the AR and MR categories, while the Z-axis indicates the corresponding metric values. Notably, the True Accepts metric significantly dominates the other categories, with values of 235 and 232 for AR and MR respectively, highlighting the high success rate of user authentications. False Rejects, False Accepts, and True Rejects exhibit much lower values, indicating improved system reliability and security.

**Table 2:** Comparative analysis of biometric authentication performance.

Method	FAR (%)	FRR (%)	EER (%)	Auth latency (ms)
Face	2.10	4.50	2.80	410
Fingerprint	1.70	3.90	2.10	450
Gaze biometric	2.80	5.10	3.20	420
Proposed (AR)	**0.27**	**0.81**	**0.53**	**530**
Proposed (MR)	**0.45**	**0.92**	**0.68**	**480**

### Low-resource performance evaluation

To assess the system feasibility on lower-end hardware, we tested the AR application on a 2018 Samsung Galaxy Tab A (Snapdragon 450 processor, 3GB RAM, Adreno 506 GPU, running Android 10). Despite limited hardware capabilities, the application remained functional, albeit with increased loading and rendering times.

Biometric authentication was completed within approximately 850–1100 ms, while 3D model rendering latency increased to around 400 ms (compared to 10–200 ms on modern hardware). Nevertheless, interactive features such as zooming and rotation were preserved, with an average frame rate of approximately 25 FPS. These findings suggest that while optimal performance is achieved on mid-to-high-end devices, the system remains usable and interactive on older hardware through practical optimizations such as mesh simplification and texture downsampling, enabling broader deployment across diverse clinical settings.

### User study and feedback analysis

A structured user study was conducted with 40 medical professionals, including neurosurgeons, radiologists, and surgical assistants, to evaluate the usability, security, and clinical effectiveness of the proposed biometric-based authentication system for XR. Precisely, the medical professionals were required to fill a survey questionnaire after using the two proposed frameworks for 3D medical data visualizations via AR and MR, where the questionnaire covered the following aspects:

- *Security Perception*: How confident were you that the proposed framework can prevent unauthorized access to patient data?
- *Ease of Use*: How intuitive was the authentication process for non-technical medical staff?
- *Clinical Utility*: How effective was the XR-based 3D visualization in supporting surgery planning and execution?
- *Authentication Speed*: How efficient was the authentication process in maintaining smooth clinical workflows?
- *User Experience*: How would you rate the overall usability and comfort of AR/MR biometric authentication?
- *Privacy Concerns*: How confident are you that the system ensures data security and complies with GDPR and HIPAA regulations?
- *System Reliability*: How stable and reliable was the authentication mechanism under clinical conditions?
- *Learning Curve*: How easy was it to adopt and use the system for the first time?
- *Trust in Blockchain*: How much do you trust blockchain technology for securing biometric data storage?

#### Key insights from medical professionals’ feedback

**Fig. 13:**
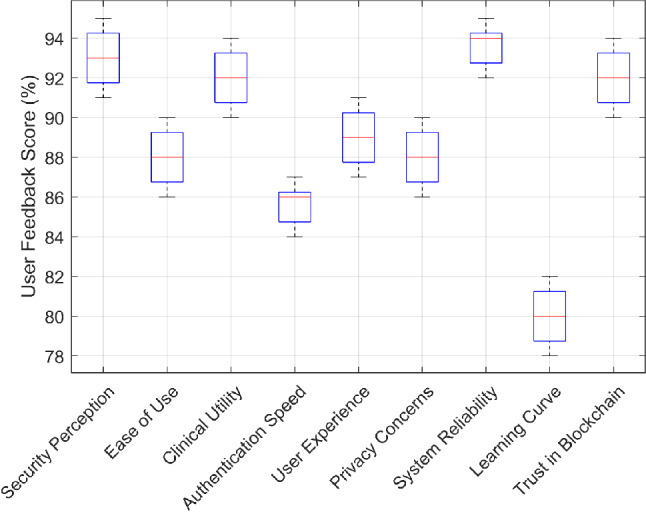
User Study and Feedback from Medical Professionals.

Figure 13 illustrates the distribution of user satisfaction scores across various evaluation metrics. The results indicate positive reception, particularly regarding security, system reliability, and trust in blockchain, which scored above 90%. Such strong confidence in the biometric authentication system stems from the ability of the proposed framework to safeguard sensitive medical records, with blockchain-based decentralized storage perceived as a significant enhancement for privacy compliance. Further, the medical professionals appreciated the practicality and ease-of-use of gaze-based authentication (average score of 85%), highlighting its intuitive integration into their existing workflow and real-world clinical environments. On similar lines, clinical utility received a high rating of 91%, with doctors emphasizing the usefulness of real-time XR visualization for surgical planning and intra-operative reference. Many respondents noted that the interactive 3D brain MRI visualization improved spatial understanding of tumor regions and facilitated better surgeon-patient communication.

On the other hand, ease of use was rated 87%, indicating that most professionals found the system intuitive. However, variability in responses suggests that some users required a brief onboarding period. Similarly, the learning curve scored 81%, highlighting that a minority of participants needed additional training. While privacy concerns were rated 88%, reflecting high trust in biometric data security through blockchain storage, a small subset of respondents (around 12%) raised concerns about long-term data retention policies. This highlights the need for granular access control and user-defined data retention periods. Additionally, authentication speed was perceived as sufficient, with an average biometric verification time of 480 to 530 ms. While this was deemed acceptable for routine medical workflows, some surgeons suggested further reducing latency to ensure usability in time-sensitive emergency situations.

Overall, medical professionals found the XR-based authentication system to be highly secure, clinically useful, and reliable, with minor recommendations for usability improvements.

The structured user study involving 40 medical professionals was conducted in accordance with the relevant ethical guidelines and institutional policies of the Indian Institute of Technology Jodhpur. No formal human ethical clearance was required for this study, as no experimental interventions involving human subjects were performed. The participants were only asked to provide user feedback and were not involved in any experimental procedures. Informed consent was obtained from all participants prior to their involvement. Additionally, this study utilized anonymized, previously collected medical imaging data from the ReMIND database^20^, and did not involve any new data collection from human subjects. Therefore, formal ethical approval was not required according to the institutional policies of IIT Jodhpur.

### Comparative analysis

A deeper statistical analysis of the performance of the proposed framework reveals that a reduction is observed in Retrieval Time, i.e., cached retrieval improves performance by 98.57% compared to uncached IPFS retrieval. Additionally, an overall performance improvement of 66.45% is observed in the system due to caching. Furthermore, it is observed that a reduction in the biometric authentication latency improves overall system response by 71.43%.

Next, a detailed comparison of the hybrid approach (a combination of Leveled HE and FV), standalone FV, and standalone Leveled HE is presented in Table 3, focusing on key aspects such as security, computational efficiency, and applicability. It can be observed from Table 3 that the hybrid architecture provides a reliable trade-off to very highly secure but least efficient Leveled HE and moderately secure but highly efficient FV. Similar behavior can also be observed for other key parameters as well. In conclusion, the hybrid approach combines the strengths of both FV and HE, making it suitable for high-security and privacy-sensitive applications, such as XR-based medical data visualization and GDPR-compliant healthcare systems.

Table 4 provides a comparative analysis between the proposed decentralized XR healthcare framework and two existing systems developed by Qayyum et al.^24^ and Kang et al.^25^. The comparison highlights key features across multiple dimensions, including XR support, biometric authentication, blockchain redactability, storage architecture, compliance with data protection regulations (GDPR/HIPAA), threat mitigation capabilities, clinical deployment status, validation methodology, and real-world applicability. The proposed framework is the only one to support biometric authentication, redactable blockchain, and full regulatory compliance, while also demonstrating successful deployment in a clinical setting using Android and HoloLens 2. It further distinguishes itself by mitigating a wide range of security threats (e.g., replay, MitM, spoofing) and validating its design through a user study involving 40 clinicians. In contrast, both referenced systems remain largely conceptual or simulated, with no evidence of real-world deployment or user-based validation. This table underscores the comprehensive nature and practical maturity of the proposed system compared to prior work.

**Table 3:** Comparison of Hybrid (a combination of Leveled HE and FV), standalone FV, and standalone Leveled HE.

Aspect	Hybrid (Leveled HE + FV)	FV	Leveled HE
Security	High	Moderate	Very high
Error tolerance	High	High	None
Computational overhead	Moderate	Low	High
Privacy during computation	Very high	Low	Very high
Storage complexity	Moderate	Low	High
Scalability	High	Moderate	Low
Resistance to attacks	Very high	Moderate	High
Regulatory compliance	High	Moderate	High
Energy efficiency	Moderate	High	Low
Implementation Complexity	High	Low	High
Use cases	High-security applications	Lightweight biometric systems	Privacy-preserving computation

**Table 4:** Comparison of the proposed framework with existing decentralized XR healthcare systems.

Feature	Proposed framework	Qayyum et al.^24^	Kang et al.^25^
XR Support	✓	✓	✓
Biometric Authentication	✓	✗	✗
Redactable Blockchain	✓	✗	✗
Decentralized Storage	IPFS + Edge Caching (✓)	✗	Hierarchical cross-chain (✓)
GDPR/HIPAA Compliance	Full	✗	Partial
Security Threat Mitigation	Replay, MitM, Spoofing, etc. (✓)	Conceptual discussion only	Focus on privacy and freshness
Clinical Deployment	Android + HoloLens 2 (✓)	✗	✗
Validation	User study (40 clinicians)	Conceptual only	Simulation only
Real-World Use	Fully implemented and tested	No real deployment	No real deployment

## Security analysis

This section presents a focused security analysis of the proposed decentralized privacy-preserving XR system for 3D medical data visualization. It evaluates the system’s robustness against the threats identified in Section “Threat model” and illustrated in Fig. 2. The analysis emphasizes cryptographic principles such as irreversibility, unlinkability, and data integrity in compliance with ISO/IEC 24745:2022(E). Each identified threat is addressed with dedicated countermeasures supported by mathematical formalization.

### Core security properties and technical countermeasures

Firstly, we present the core security properties needed in such systems and how our proposed framework ensures them.

#### Irreversibility of biometric data

To ensure irreversibility and protect biometric data from reconstruction attacks, the framework uses a combination of leveled HE and FV mechanisms:

1 $$\begin{aligned} \textbf{E}_1 = \text {encryptLHE}(\{w_1, w_2, \ldots , w_n\}, K) \end{aligned}$$

The probability of reconstructing the original template without the key is:

2 $$\begin{aligned} P_{\text {reconstruct}} = \frac{1}{2^n} \end{aligned}$$

where *n* is the number of chaff points, exponentially reducing the feasibility of brute-force attempts.

#### Unlinkability of biometric templates

To prevent linking of biometric data across sessions, the system incorporates randomized chaff vectors per session:

3 $$\begin{aligned} I(w_i; R) \approx 0 \end{aligned}$$

This ensures minimal mutual information between genuine and chaff data, satisfying unlinkability.

#### Replay attack resistance

Replay attacks are mitigated using session-specific nonces within dynamic challenge-response authentication:

4 $$\begin{aligned} H_{\text {auth}}(B_i, t)= & H(B_i) \oplus r(t) \end{aligned}$$

5 $$\begin{aligned} P_{\text {replay}}= & \frac{1}{2^n} \end{aligned}$$

6 $$\begin{aligned} H(r(t))= & -\sum _{i=1}^{m} p_i \log _2 p_i \end{aligned}$$

These mechanisms ensure freshness and prevent reuse of intercepted credentials.

#### Biometric template reconstruction attacks

Even if vaults are leaked, template reconstruction is infeasible due to encrypted RS-coded features and chaff obfuscation:

7 $$\begin{aligned} P_{\text {reconstruct}}= & \frac{1}{2^m} \end{aligned}$$

8 $$\begin{aligned} H(B)= & -\sum _{i=1}^{k} p_i \log _2 p_i \end{aligned}$$

where *m* is the number of obfuscating points and *H*(*B*) denotes entropy of biometric templates.

#### Man-in-the-middle (MitM) attacks

To ensure resistance against MitM attacks, all communication between XR clients and backend services is secured via encrypted transport protocols. Biometric data is encrypted using leveled HE before transmission, ensuring confidentiality even if intercepted. Additionally, session-specific tokens are incorporated to prevent reuse and enhance freshness of authentication.

$$\sigma = H(E) \oplus T_s$$

where $E = \text {encryptLHE}(\{w_1, w_2, \dots , w_n\}, K)$ is the encrypted biometric payload and $T_s$ is the time-variant session token, ensuring replay protection and transmission security.

#### Denial-of-service (DoS) attacks

To mitigate DoS attacks, the framework utilizes edge caching for frequently accessed 3D medical data, significantly reducing load on IPFS and improving system responsiveness. Authentication is performed on edge devices, and access logic is distributed via hierarchical blockchain layers, limiting centralized bottlenecks. Failed authentications are rate-limited to prevent brute-force flooding.

$$\Delta C = C_{\text {ipfs}} - C_{\text {cached}}, \quad \text {with } C_{\text {cached}} \ll C_{\text {ipfs}}$$

This caching strategy enhances availability under load while ensuring uninterrupted access to critical patient visualizations.

#### Data tampering and unauthorized blockchain modification

Hierarchical redactable blockchain with chameleon hash-based redactions ensures verifiable and authorized modifications only:

9 $$\begin{aligned} H(\text {File})= & \text {SHA-256}(\text {File Content}) \end{aligned}$$

10 $$\begin{aligned} H_{\text {chameleon}}(m, r)= & H(m) \oplus r_{\text {trapdoor}} \end{aligned}$$

11 $$\begin{aligned} P_{\text {tamper}}= & \frac{1}{2^{\lambda /2}}, \quad \lambda = 256 \end{aligned}$$

This provides strong auditability and guarantees immutability unless redactions are properly authorized.

### Medical data security challenges and mitigation strategies

3D medical data visualization introduces unique privacy and security demands. The framework addresses these as follows:

#### High sensitivity of medical data

Medical information is encrypted using leveled HE even during computation, preventing any unauthorized inference.

#### Complex data sharing requirements

Blockchain-based access control ensures data sharing is authorized and logged, facilitating institutional collaboration and traceability.

#### Real-time access with secure storage

Edge caching accelerates access while IPFS ensures secure, decentralized storage. Periodic hash verification ensures data integrity.

#### Regulatory compliance in healthcare

To ensure regulatory compliance, the proposed framework incorporates privacy-preserving mechanisms aligned with the GDPR and HIPAA. GDPR compliance is achieved through redactable blockchain-based access control that enforces the “right to be forgotten” through consensus-based redactions and supports data traceability^5,11^. HIPAA compliance is addressed by implementing encryption for data-at-rest and in-transit using leveled HE, audit logging via smart contracts, and decentralized authentication^10^. These features collectively ensure confidentiality, integrity, and accountability of sensitive health information in line with international standards within the proposed XR healthcare system.

## Conclusion

In this work, a decentralized and secure XR framework has been proposed for 3D visualization of medical data, specifically tailored for remote surgical planning and diagnosis. A Hybrid Biometric Cryptosystem (HBC), hierarchical redactable blockchain, and IPFS-based decentralized storage were integrated to address challenges associated with centralized architectures, namely data privacy, access control, and visualization quality. Through the proposed framework, a scalable system has been established that enables secure, collaborative, and immersive interaction with sensitive medical data. The framework ensures compliance with healthcare data regulations. GDPR alignment is achieved via redactable blockchain mechanisms supporting data erasure and auditability. Additionally, HIPAA compliance is addressed through encryption of patient and biometric data, secure session protocols, and detailed access logging via smart contracts. These safeguards support confidentiality, integrity, and traceability while maintaining the real-time, high-fidelity interaction essential for clinical deployment. Additionally, edge caching has been incorporated to improve data retrieval efficiency, significantly enhancing system responsiveness and enabling real-time interaction with large 3D datasets in XR environments. The system was implemented and validated using both AR and MR platforms, via an Android application and Microsoft HoloLens 2, respectively. Its effectiveness and clinical relevance were further reinforced through structured user studies involving 40 healthcare professionals. The outcomes demonstrated significant improvements in spatial understanding of anatomical regions such as tumors, and positive feedback was received regarding its usability, performance, and regulatory alignment. Overall, the proposed framework provides a promising solution for secure, collaborative, and efficient medical data visualization in digital healthcare. Future work will focus on extending the platform to support real-time, XR-based teleoperative surgeries.

## Data Availability

This study uses publicly available data from the ReMIND database (https://www.cancerimagingarchive.net/collection/remind/), which is cited in the manuscript. No new datasets were generated or analyzed during the current study. All data supporting the findings are included in the manuscript.
